# Association of Human TLR1 and TLR6 Deficiency with Altered Immune Responses to BCG Vaccination in South African Infants

**DOI:** 10.1371/journal.ppat.1002174

**Published:** 2011-08-11

**Authors:** April Kaur Randhawa, Muki S. Shey, Alana Keyser, Blas Peixoto, Richard D. Wells, Marwou de Kock, Lesedi Lerumo, Jane Hughes, Gregory Hussey, Anthony Hawkridge, Gilla Kaplan, Willem A. Hanekom, Thomas R. Hawn

**Affiliations:** 1 Department of Medicine, University of Washington School of Medicine, Seattle, Washington, United States of America; 2 South African Tuberculosis Vaccine Initiative, Institute of Infectious Diseases and Molecular Medicine and School of Child and Adolescent Health, University of Cape Town, South Africa; 3 Public Health Research Institute, University of Medicine and Dentistry of New Jersey, Newark, New Jersey, United States of America; Harvard School of Public Health, United States of America

## Abstract

The development of effective immunoprophylaxis against tuberculosis (TB) remains a global priority, but is hampered by a partially protective Bacillus Calmette-Guérin (BCG) vaccine and an incomplete understanding of the mechanisms of immunity to *Mycobacterium tuberculosis.* Although host genetic factors may be a primary reason for BCG's variable and inadequate efficacy, this possibility has not been intensively examined. We hypothesized that Toll-like receptor (TLR) variation is associated with altered *in vivo* immune responses to BCG. We examined whether functionally defined TLR pathway polymorphisms were associated with T cell cytokine responses in whole blood stimulated *ex vivo* with BCG 10 weeks after newborn BCG vaccination of South African infants. In the primary analysis, polymorphism TLR6_C745T (P249S) was associated with increased BCG-induced IFN-γ in both discovery (n = 240) and validation (n = 240) cohorts. In secondary analyses of the combined cohort, TLR1_T1805G (I602S) and TLR6_G1083C (synonymous) were associated with increased IFN-γ, TLR6_G1083C and TLR6_C745T were associated with increased IL-2, and TLR1_A1188T was associated with increased IFN-γ and IL-2. For each of these polymorphisms, the hypo-responsive allele, as defined by innate immunity signaling assays, was associated with increased production of TH1-type T cell cytokines (IFN-γ or IL-2). After stimulation with TLR1/6 lipopeptide ligands, PBMCs from TLR1/6-deficient individuals (stratified by TLR1_T1805G and TLR6_C745T hyporesponsive genotypes) secreted lower amounts of IL-6 and IL-10 compared to those with responsive TLR1/6 genotypes. In contrast, no IL-12p70 was secreted by PBMCs or monocytes. These data support a mechanism where TLR1/6 polymorphisms modulate TH1 T-cell polarization through genetic regulation of monocyte IL-10 secretion in the absence of IL-12. These studies provide evidence that functionally defined innate immune gene variants are associated with the development of adaptive immune responses after *in vivo* vaccination against a bacterial pathogen in humans. These findings could potentially guide novel adjuvant vaccine strategies as well as have implications for IFN-γ-based diagnostic testing for TB.

## Author Summary

Tuberculosis (TB) is one of the leading infectious causes of death worldwide. The current vaccine for TB, BCG, is widely used but it is not highly effective in preventing disease. We investigated the role of host genetics in the immune response to BCG vaccination. We found that variants of innate immunity genes (*TLR1* and *TLR6*) were associated with BCG-induced immune responses after vaccination. These findings may guide new strategies for vaccine development as well as diagnosis of TB.

## Introduction

Tuberculosis (TB) is one of the leading causes of mortality worldwide, with 1.7 million deaths occurring annually [1]. Although murine studies have uncovered numerous genes and pathways which are critical for effective immune responses to *Mycobacterium tuberculosis* (Mtb), the mechanisms of immune regulation in humans are largely unknown. The current TB vaccine, Bacillus Calmette-Guérin (BCG), is widely used and has been available since 1921, but provides partial and inconsistent protection [2], [3]. Several hypotheses have been suggested to explain BCG's inconsistent efficacy including differences among BCG vaccine strains, modulation of immune responses by previous exposure to environmental mycobacteria, and host genetics [4], [5], [6]. Evidence presented in numerous studies over the past 50 years suggests that host genetics influences susceptibility to TB. First, twin studies indicate that TB rates among monozygotic twins are more than twice the rate of dizygotic twins [7]. Second, genome-wide linkage and association studies (GWAS) have identified several susceptibility loci and efforts to identify the implicated genes are ongoing [8], [9], [10], [11]. Third, candidate gene association studies have reported several loci associated with TB susceptibility and some of these have been replicated and/or involved polymorphisms with well-characterized function [10]. In addition to susceptibility to active TB disease, host genetic factors may regulate tuberculin skin reactivity after Mtb exposure or BCG vaccination [12], [13], [14]. In addition, IFN-γ and TNF cytokine responses after *ex vivo* stimulation of blood or PBMCs with BCG or Mtb may be genetically controlled [15], [16], [17] Although there is wide variation in IFN-γ levels after BCG vaccination, the genetic mechanisms which regulate these differences are unknown [18], [19]. Understanding these differences may illuminate mechanisms of protective immunity to TB. Furthermore, clinical diagnoses of TB commonly utilize purified protein derivative PPD skin testing, which relies on host release of interferon-γ (IFN-γ) to induce a positive result. Host factors could confound PPD testing if results are regulated by genetic variation in IFN-γ responses that differ between individuals. To our knowledge, no studies have examined whether genetic variants are associated with immunologic or clinical endpoints after BCG vaccination.

Human TLRs are a family of 10 proteins that differentially recognize pathogen-associated molecular patterns (PAMPs) and activate signaling cascades that lead to initiation of the innate immune response and cytokine production, ultimately culminating in antimicrobial host defenses. Several genes in the TLR pathway mediate recognition of Mtb including TLRs1, 2, 4, 6, and 9, which interact with the adaptor proteins MyD88 and TIRAP/Mal to activate macrophages and dendritic cells [20], [21]. In addition to a central role in regulation of innate immunity, murine studies have shown that TLRs modulate the development of T cell responses through effects on dendritic cells and antigen presentation [22], [23], [24]. Vaccination studies demonstrate that distinct T cell responses are stimulated when the TLR-ligand adjuvant is varied. For example, vaccination of mice with ovalbumin with a TLR4 ligand generates a Th-1 ova-specific response, whereas ovalbumin with TLR1/2/6 ligands generates a Th2 response [25]. It is not currently known if TLRs affect vaccination outcomes in humans and/or whether the type of modulation is similar to mice.

Human immunology studies have been hampered by population and experimental heterogeneity, which often limit the level of biologic insight. We and others have discovered single nucleotide polymorphisms (SNPs) and mutations in the TLR pathway that regulate cellular function and are associated with susceptibility to some infections, including TB. Hypofunctional polymorphisms have been reported in *TLR1* (T1805G), *TLR2* (C597T and G2258A), *TLR4* (A896G and C1196T), *TLR6* (C745T and G1083C), and *TIRAP/MAL* (C539T, G558T) [20], [21], [26], [27], [28], [29], [30], [31], [32], [33], [34]. These TLR-deficient humans offer experimental advantages that are similar to the characterization of TLR-deficient mice generated with gene knockout strategies, yet avoid the limitations of mouse models. Although these polymorphisms regulate NF-κB signaling in transfected cells and/or are associated with deficient signaling in primary monocytes, it is not currently known if these or any TLR variants are associated with *in vivo* vaccine responses and/or T-cell responses.

We hypothesized that common variants of TLR pathway genes are associated with *in vivo* BCG-induced immune responses. To identify immunogenetic correlates of vaccine responsiveness, we examined whether functionally defined polymorphisms in TLR pathway genes are associated with BCG-induced immune responses 10 weeks after vaccination of infants.

## Results

### Primary analysis: polymorphism TLR6_C745T is associated with increased IFN-γ production after BCG vaccination

**Figure 1:**
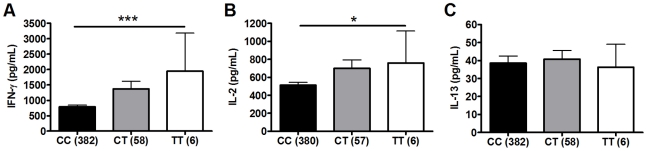
Polymorphism TLR6_C745T is associated with BCG-induced whole blood cytokine production. Whole blood drawn 10 weeks after BCG vaccination of newborns was re-stimulated with BCG for 7h *ex vivo* and plasma levels of IFN-γ, IL-2, and IL-13 were measured. A general linear model was used to examine whether polymorphism TLR6_C745T was associated with BCG-induced cytokine levels after subtraction of unstimulated control values. IFN-γ (A), IL-2 (B), and IL-13 (C) levels are shown with unstimulated control values subtracted for each genotype of polymorphism TLR6_C745T. The number of individuals for each genotype is shown in parentheses. P values represent significant differences as assessed using the general linear model. *P<0.05, **P<0.01, ***P≤0.001.

To investigate whether polymorphisms in innate immunity genes are associated with vaccine-induced immune responses, we enrolled South African infants routinely vaccinated with BCG at birth. Ten weeks after vaccination, we stimulated whole blood *ex vivo* with BCG and measured plasma levels of Th1 (IFN-γ and IL-2) and Th2 (IL-13) T cell cytokines. We examined whether 9 well-characterized SNPs in 5 TLR pathway genes (*TLR1, 2, 4, 6, and TIRAP/MAL*) were associated with these cytokine levels. These 9 variants are involved in regulating the immune response to Mtb and were selected due to their modulation of signaling responses, and/or association with clinical susceptibility to infection [21], [26], [27], [28], [34], [35], [36], [37], [38]. For our primary analysis, we examined associations in a discovery sample set of 240 and then validated positive results in a second sample set of 240. One polymorphism (TLR2_G2258A) was essentially invariant (one heterozygote detected) and was not analyzed further. Polymorphisms TLR6_C745T and TLR6_G1083C were associated with increased IFN-γ and IL-2, respectively, in the discovery sample set (Table 1 and Figure 1, P = 0.001 and P = 0.024, respectively, for analysis of BCG-stimulated value after subtraction of unstimulated control level). To correct for confounding effects from multiple comparisons, we analyzed the association in a validation set of 240 samples. TLR6_745T was also associated with BCG-increased IFN-γ levels in this sample set (P = 0.03). TLR6_G1083C was not associated with IL-2 in the validation data set. None of the other polymorphisms were associated with BCG-induced cytokines in this primary analysis. TLR6_C745T was not associated with BCG-induced IL-13 levels or with unstimulated control levels of any of the cytokines (Figure 1).

**Table 1:** Association of TLR-pathway polymorphisms with cytokine responses following BCG vaccination in discovery cohort.^a^

		AA			Aa			aa			
SNP	Cytokine	N	Mean	SEM	N	Mean	SEM	N	Mean	SEM	P^b^
**TIRAP_C539T**	**IFN-γ**	209	916.00	93.98	16	761.50	217.18	2	988.32	1080.70	0.796
	**IL-2**	209	566.10	50.43	16	385.50	64.55	2	330.64	349.60	0.297
	**IL-13**	210	38.90	4.11	16	26.70	4.83	2	13.65	15.70	0.324
**TIRAP_C558T**	**IFN-γ**	186	916.90	96.19	37	846.40	233.53	3	199.47	261.90	0.502
	**IL-2**	185	572.00	54.80	38	432.90	73.45	3	425.56	484.90	0.297
	**IL-13**	186	39.50	4.52	38	29.50	5.22	3	14.43	24.80	0.294
**TLR1_T1805G**	**IFN-γ**	176	840.10	89.31	45	1256.80	268.30	3	246.64	275.10	0.200
	**IL-2**	177	553.30	54.70	44	587.00	98.08	3	115.41	149.10	0.833
	**IL-13**	177	36.40	3.96	45	46.70	11.25	3	12.82	16.40	0.521
**TLR2_C597T**	**IFN-γ**	83	765.20	128.65	110	981.30	133.13	32	262.99	1056.40	0.214
	**IL-2**	82	484.40	57.50	111	617.80	81.07	32	94.36	522.30	0.506
	**IL-13**	83	28.20	2.76	111	45.30	7.19	32	7.04	39.30	0.138
**TLR4_A896G**	**IFN-γ**	202	937.00	91.52	23	694.80	331.02	-	-	-	0.409
	**IL-2**	202	564.30	50.57	23	479.40	125.05	-	-	-	0.586
	**IL-13**	203	38.90	4.17	23	31.60	8.09	-	-	-	0.563
**TLR4_C1196T**	**IFN-γ**	215	896.90	86.22	11	892.20	678.34	-	-	-	0.991
	**IL-2**	215	552.00	47.99	11	458.60	223.33	-	-	-	0.668
	**IL-13**	216	37.50	3.93	11	39.70	16.37	-	-	-	0.905
**TLR6_G1083C**	**IFN-γ**	106	822.60	127.80	104	883.60	115.62	15	536.51	1744.70	0.070
	**IL-2**	107	436.80	48.61	103	661.10	85.04	15	172.48	678.30	**0.024**
	**IL-13**	107	32.90	3.44	104	44.00	7.34	15	11.39	35.60	0.320
**TLR6_C745T**	**IFN-γ**	191	815.00	85.72	32	1219.70	274.91	3	2199.76	3606.30	**0.001**
	**IL-2**	192	532.20	51.30	31	614.40	113.04	3	626.08	1225.50	0.165
	**IL-13**	192	37.20	4.34	32	41.80	7.14	3	23.09	46.70	0.625
											

a Whole blood from 10-week old infants vaccinated at birth with BCG was re-stimulated with BCG ex vivo for 7 hours and plasma levels of IFN-γ, IL-2, and IL-13 were measured in a discovery cohort sample set (n = 240).

b P value calculated from a general linear model that examined whether TLR polymorphisms were associated with BCG-induced cytokine levels after subtraction of unstimulated control values.

We next performed a subgroup analysis to determine whether the association was affected by population stratification. TLR6_C745T was associated with increased IFN-γ and IL-2 levels in the South African Mixed Ancestry subgroup (Table S1, P = 0. in combined dataset with n = 371 individuals). We further examined this subgroup for evidence of population admixture by examining 21 polymorphisms which were previously selected as ancestry informative markers in South Africa [39]. We compared the frequencies of these 21 polymorphisms in groups of low (less than the median) and high (greater than the median) cytokine responses. For IFN-γ, IL-2, and IL-13, there were no significant differences in the frequencies of the polymorphisms when comparing the stratified groups (Table S2, global P values of 0.40, 0.38, and 0.39 for IFN-γ, IL-2, and IL-13, respectively). These data suggested that low and high cytokine responses were not attributable to population admixture. Together, these results suggest that TLR6_745T, a hypofunctional polymorphism in monocyte assays, was associated with increased levels of IFN-γ after BCG vaccination.

### Secondary analysis: polymorphisms TLR6_G1083C and TLR1_T1805G are associated with BCG-induced cytokines

**Figure 2:**
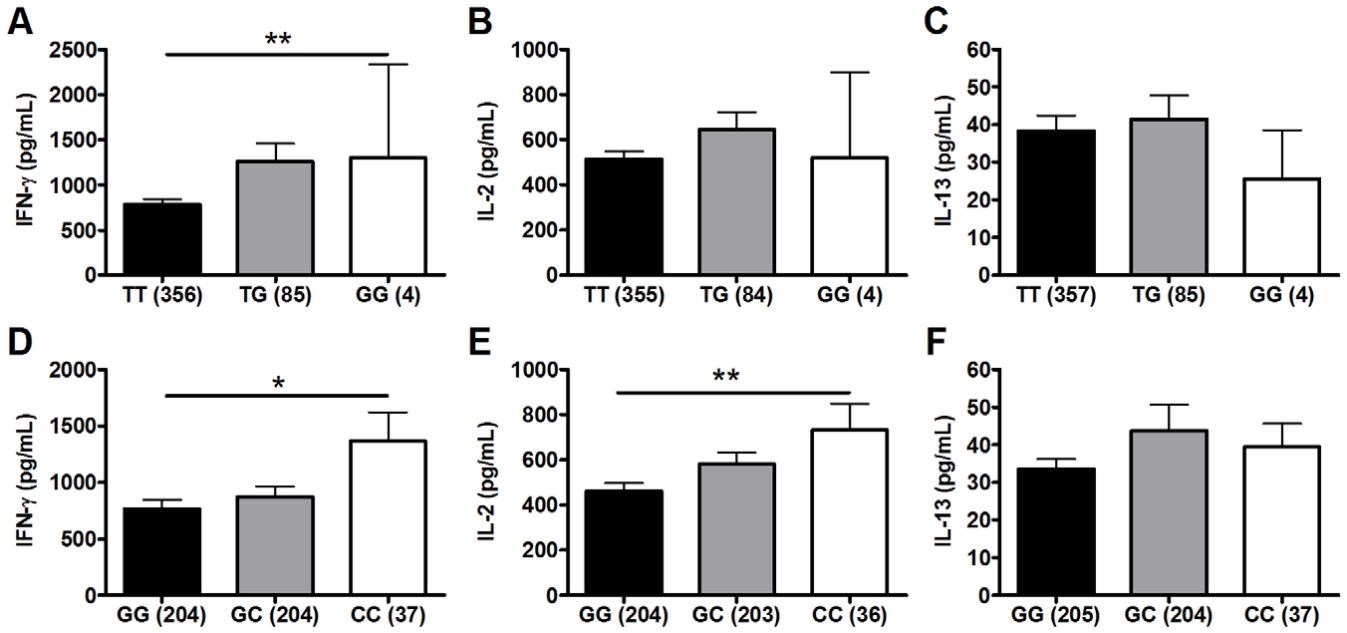
Polymorphisms TLR6_G1083C and TLR1_T1805G are associated with BCG-induced whole blood cytokine production. Whole blood drawn 10 weeks after BCG vaccination of infants was re-stimulated with BCG for 7h *ex vivo* and plasma levels of IFN-γ, IL-2, and IL-13 were measured. A general linear model was used to examine whether TLR polymorphisms were associated with BCG-induced cytokine levels after subtraction of unstimulated control values. IFN-γ, IL-2, and IL-13 levels are shown with unstimulated control values subtracted for each genotype of polymorphism TLR1-T1805G (A–C) and TLR6_G1083C (D–F). The number of individuals for each genotype is shown in parentheses. P values represent significant differences as assessed using the general linear model. *P<0.05, **P<0.01.

We next performed a secondary analysis of the combined dataset (n = 480) to increase the power to detect associations of low frequency polymorphisms with cytokine responses. For low frequency polymorphisms (minor allele ≤10%, n = 5), we examined whether variants were associated with BCG-induced cytokines. One additional polymorphism, TLR1_T1805G was associated with increased IFN-γ production in the combined dataset (Table 2 and Figure 2). The hypofunctional G allele was associated with higher IFN-γ production, but not IL-2, (Figure 2 and Table 2, P = 0.002). Due to the initial association of TLR6_G1083C with IL-2 in the discovery dataset, we re-examined its association in the combined dataset. The hypofunctional C allele was associated with higher IFN-γ and IL-2 production in the combined dataset (P = 0.021 and 0.008 for IFN-γ and IL-2, respectively, Figure 2 and data not shown). In addition, TLR6_C745T was associated with IL-2 production (Figure 1, P = 0.031). There were no associations with IL-13 production for any of these SNP. Each of these analyses was also validated in population subgroups to ensure there was no confounding from population admixture (Table S1). The other 4 polymorphisms were not associated with IFN-γ, IL-2, or IL-13 levels (Table 2). We used a Bonferroni correction for multiple comparisons in the secondary analysis due to the absence of a validation sample set for this analysis. The association of TLR1_T1805G with IFN-γ remained significant after a Bonferroni correction for multiple comparisons (P = 0.002×15 (3 cytokine and 5 polymorphisms)  = 0.03). The association of TLR6_G1083C with IL-2 was not significant after correction. Together, these results suggest that TLR1/6 hypofunctional innate immune gene polymorphisms (TLR6_745T and TLR1_1805G with statistical validation; TLR6_1083C without validation) are associated with increased IFN-γ or IL-2 levels after BCG vaccination.

**Table 2:** Secondary analysis of association of TLR polymorphisms with BCG-induced cytokines in combined dataset.^a^

		MAFb	AA			Aa			aa			
SNP	Cytokine		N	Mean	SEM	N	Mean	SEM	N	Mean	SEM	Pc
**TIRAP_C539T**	**IFN-γ**	0.041	414	878.80	64.54	31	813.90	158.29	3	971.20	581.05	0.871
	**IL-2**		412	539.50	32.53	31	512.80	82.89	3	499.80	242.89	0.809
	**IL-13**		415	36.50	2.45	31	67.50	37.97	3	21.10	9.53	0.086
**TIRAP_C558T**	**IFN-γ**	0.078	377	897.40	66.83	61	787.10	161.01	4	216.70	148.10	0.307
	**IL-2**		374	550.50	34.61	62	463.60	64.83	4	390.30	315.45	0.281
	**IL-13**		377	37.10	2.61	62	49.60	19.34	4	19.90	11.30	0.382
**TLR1_T1805G**	**IFN-γ**	0.104	356	783.90	57.22	85	1256.70	202.85	4	1298.00	1037.70	**0.002**
	**IL-2**		355	513.40	33.65	84	645.70	76.22	4	519.40	379.15	0.128
	**IL-13**		357	38.40	4.07	85	41.40	6.32	4	25.60	12.95	0.866
**TLR4_A896G**	**IFN-γ**	0.043	407	878.20	62.39	38	745.90	222.60	0	-	-	0.539
	**IL-2**		405	547.00	32.77	38	442.30	80.49	0	-	-	0.341
	**IL-13**		408	39.00	3.73	38	35.20	7.61	0	-	-	0.759
**TLR4_C1196T**	**IFN-γ**	0.023	424	876.70	61.53	20	806.20	371.37	0	-	-	0.811
	**IL-2**		422	540.60	31.71	20	430.90	124.12	0	-	-	0.459
	**IL-13**		425	38.70	3.62	20	37.90	9.21	0	-	-	0.965

a Whole blood from 10-week old infants vaccinated at birth with BCG was re-stimulated with BCG ex vivo for 7 hours and plasma levels of IFN-γ, IL-2, and IL-13 were measured in a combined sample set (n = 480).

b Abbreviations: MAF, minor allele frequency.

c P value calculated from a general linear model that examined whether TLR polymorphisms were associated with BCG-induced cytokine levels after subtraction of unstimulated control values.

### TLR1_A1188T, a TLR6/1/10 haplotype tagging polymorphism, is associated with increased IFN-γ and IL-2 after BCG stimulation

**Figure 3:**
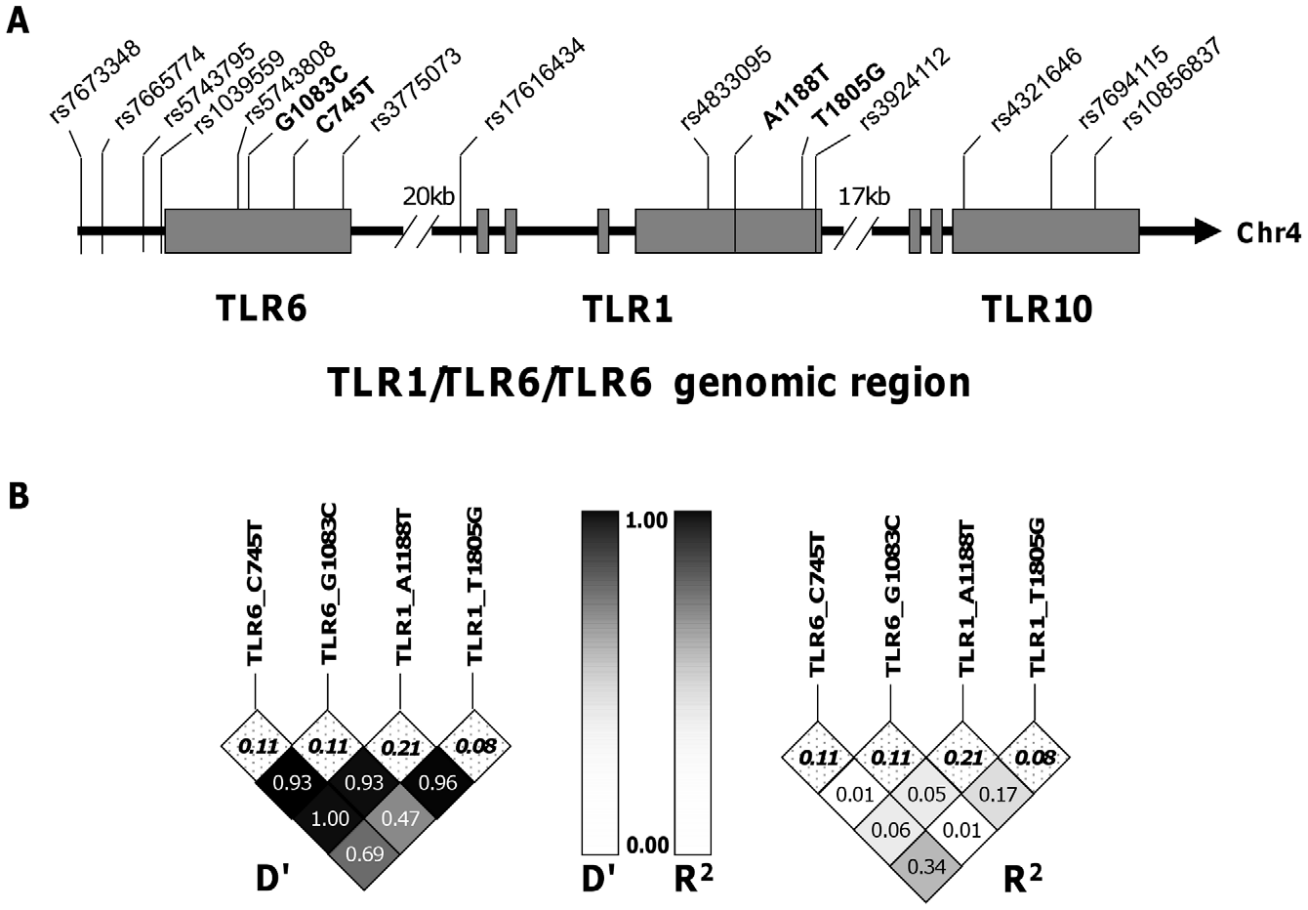
Haplotype tagging SNPs and pair-wise linkage disequilibrium analysis of TLR1-6-10 gene region. A. The arrangement of TLR1 TLR6 and TLR10 on chromosome 4 and the position of the haploytype-tagging SNPs are shown. B. Linkage disequilibrium analyses between the four TLR SNPs with functional associations in this study are expressed as D' and R^2^ for pair-wise comparisons. The minor allele frequencies (MAF) are listed in the top row of boxes and highlighted in bold.

**Figure 4:**
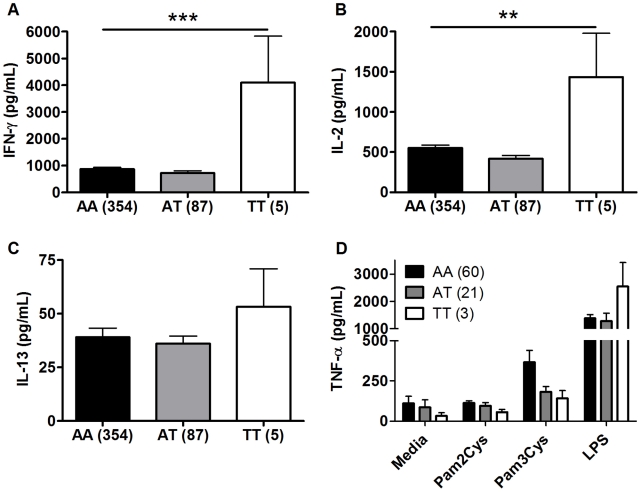
TLR1 polymorphism A1188T is associated with increased Th1 cytokine production in response to BCG stimulation. A-C. As in Figure 1, whole blood drawn 10 weeks after BCG at birth was re-stimulated with BCG for 7h *ex vivo*. Plasma IFN-γ (A), IL-2 (B), and IL-13 (C) levels are shown for individuals with TLR1 polymorphism A1188T (rs3923647) with the number of individuals for each genotype shown in parentheses. **D.** PBMCs from individuals vaccinated with BCG were stimulated with media or TLR ligands (LPS (100 ng/mL), PAM_2_CSK_4_ (250 ng/mL), PAM_3_CSK_4_ (250 ng/mL)) for 24 hours. TNF-α production was quantified by ELISA. **P<0.01, ***P≤0.001.

TLR1 and TLR6 are located on chromosome 4 in a region adjacent to TLR10 in a genomic region spanning 54 kb [40] (Figure 3). To determine if any additional SNPs in this region were associated with altered adaptive immune responses to BCG stimulation, we genotyped 13 haplotype-tagging SNPs (Figure 3 and Table S3). TLR1 polymorphism A1188T (rs3923647) was associated with increased IFN-γ and IL-2 production in both discovery and validation cohorts (P<0.001 and P = 0.002, respectively for combined dataset; Figure 4A and B and Table S3). The associations were also present in a subgroup analysis of the SA Mixed Ancestry group (Table S1). There were no statistical differences in IL-13 production (Figure 4C). Previous studies suggest that the 1188T allele is associated with decreased NF-κb signaling in HEK293 cells [41]. To determine if this SNP resulted in regulated innate responses in primary cells, we examined the production of TNF-α by peripheral blood mononuclear cells in response to purified TLR ligands. Although there were no statistically significant differences in TNF-α production when comparing the 3 genotypes, there was a trend towards lower cytokine response to the TLR1 ligand, tri-acylated lipopeptide Pam3Cys (Figure 4D, n = 84, P = 0.098 vs P = 0.227 for media control, 0.277 for Pam2Cys, and 0.863 for LPS.).

### Haplotype analysis of the four TLR polymorphisms suggests independent associations with cytokine levels

To ascertain whether these cytokine associations were independent, we measured the linkage disequilibrium (LD) among the loci using pair-wise LD analysis in the South African mixed ancestry group. R^2^ was less than 0.34 for all pair-wise calculations and less than 0.1 for the majority, suggesting a low level of linkage disequilibrium between these polymorphisms (Figure 2). We next examined whether pairwise diplotype combinations of the polymorphisms were associated with further differences in IFN-γ and/or IL-2 levels. Due to several low polymorphism frequencies, only SNP G1083C could be assessed in combination with the others. We found no diplotype combinations that resulted in additive effects (data not shown). Taken together, these results suggest that each of the four SNPs individually contributes to the association with BCG-induced immune responses and that combination of these polymorphisms does not further increase the magnitude of the response.

### Two TLR polymorphisms (TLR6_C745T and TLR1_T1805G) are associated with increased expression of T cell cytotoxicity molecules

**Figure 5:**
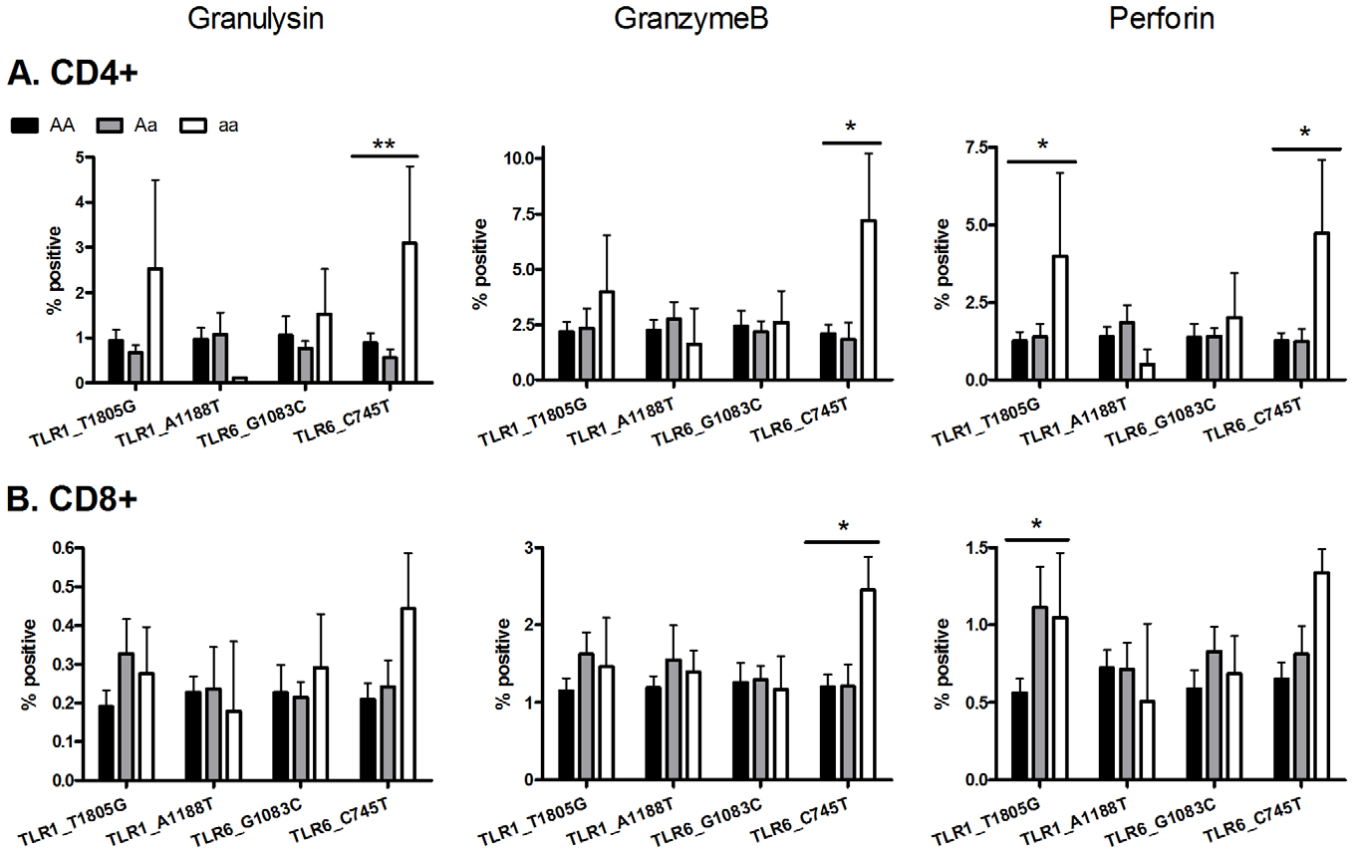
Two TLR polymorphisms are associated with increased expression of cytotoxic molecules after *ex vivo* BCG stimulation. Whole blood drawn 10 weeks after BCG at birth was re-stimulated with BCG *ex vivo* for 3 days. Granulysin, granzyme B and perforin production were measured by intracellular cytokine staining in (A) CD4-positive and (B) CD8-positive T cells. A general linear model was used to examine whether 4 TLR polymorphisms (TLR1_T1805G, TLR1_A1188T, TLR6_G1083C, and TLR6_C745T) were associated with BCG-induced cytotoxicity markers after subtraction of unstimulated control values. *P<0.05, **P<0.01.

To examine T cell function in greater detail in an exploratory analysis of the 4 polymorphisms (TLR6_C745T, TLR6_G1083C, TLR1_T1805G, TLR1_A1188T) in a subset of individuals (n = 65), we measured the expression of cytotoxic mediators granulysin, granzyme B, and perforin as well as T cell proliferation (Figure 5 and Figure S1). Due to the smaller sample set, some of the analyses had reduced power from low minor allele frequencies. TLR6 polymorphism C745T was associated with CD4-specific expression of granulysin (p<0.005) and granzyme B (p<0.05), while both TLR6_C745T and TLR1_T1805G were associated with perforin expression (p<0.02). In addition TLR6_C745T was associated with increased granzyme B, but not granulysin or perforin, in CD8+ T-cells. TLR1_T1805G was associated with increased perforin in CD8+ T-cells. In contrast, neither polymorphism affected T cell proliferation (Figure S1). When adjusted for multiple comparisons with a Bonferroni correction, these associations were not statistically significant. TLR1_A1188T and TLR6_G1083C were not associated with altered T cell function as measured by production of cytotoxic effector molecules or proliferation (Figure 5 and Figure S1). Together, these results suggest that some of the polymorphisms associated with plasma IFN-γ or IL-2 were also associated with expression of BCG-induced CD4 and CD8 T-cell cytotoxic markers.

### TLR1/6 deficiency is associated with decreased lipopeptide-induced IL-10 secretion

**Figure 6:**
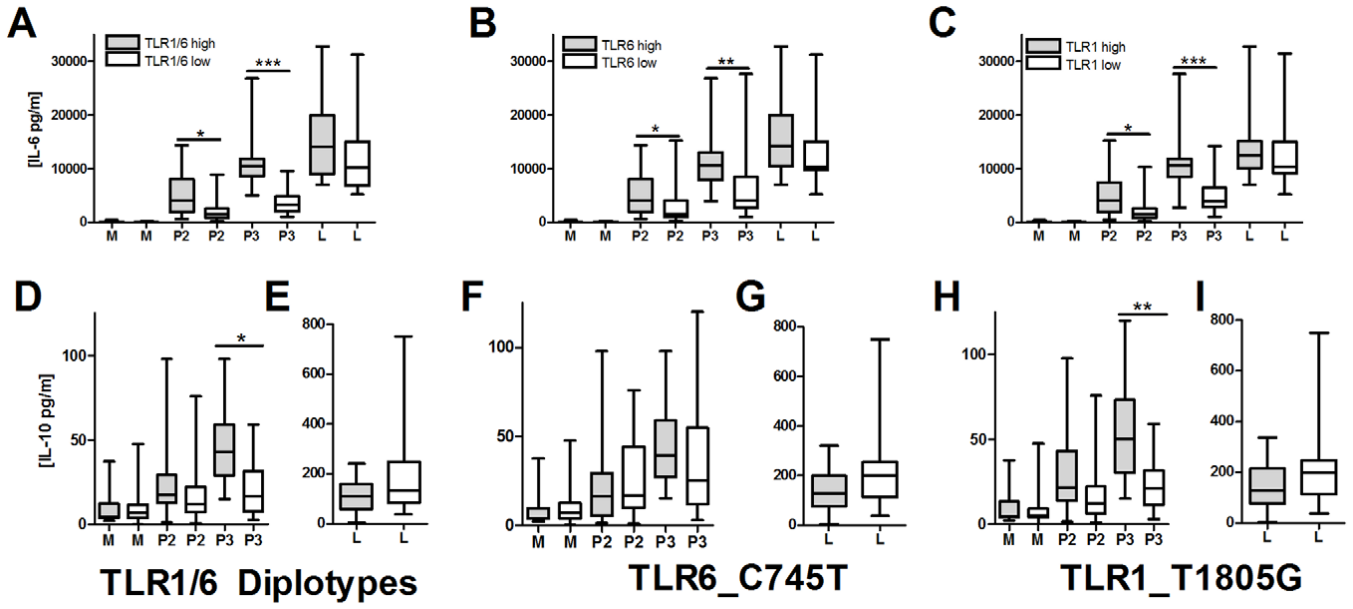
TLR1/6 polymorphisms are associated with decreased lipopeptide-induced IL-6 and IL-10 production in PBMCs. PBMCs from individuals with polymorphisms in TLR6_C745T and TLR1_T1805G were stimulated for 20 hours with TLR ligands LPS (10 ng/mL), PAM_2_CSK_4_ (250 ng/mL), or PAM_3_CSK_4_ (250 ng/mL). Supernatant IL-6 and IL-10 levels were quantified by ELISA. TLR1 and TLR6 genotypes are designated based on functionality as: TLR1_low (genotype TLR1_1805GG, n = 22), TLR1_high (1805GT/TT, n = 25), TLR6_low (TLR6_745CT/TT, n = 24), or TLR6_high (745CC, n = 23). TLR1/6 diplotypes are designated as TLR1/6_low (TLR1_1805GG and TLR6_745CT/TT, n = 18) or TLR1/6_high (TLR1_1805GT/TT and TLR6_745CC, n = 18). Panels: For IL-6, TLR1/6_low vs high diplotypes (A), TLR6_low versus high genotypes for (B), and TLR1_low versus high genotypes for IL-6 (C). For IL-10, TLR1/6_low vs high diplotypes for media, PAM2, and PAM3 (D) or LPS (E); TLR6_low versus high genotypes for media, PAM2, and PAM3 (F) or LPS (G); TLR1_low versus high genotypes for media, PAM2, and PAM3 (H) or LPS (I). Diplotypes and genotypes with normal signaling (high) designated with a box with dots while deficient signaling (low) designated with clear box. P values represent significant differences as assessed by a Mann-Whitney U-test for comparison of low versus high groups. *P<0.05, **P<0.01, ***P≤0.001.

We next examined possible mechanisms underlying the association of hypofunctional TLR1 and TLR6 polymorphisms with increased levels of BCG-induced IFN-γ from T-cells. We hypothesized that TLR1/6 polymorphisms are associated with altered T-cell responses through regulation of IL-10 and IL-12 secretion from monocytes. After stimulating PBMCs or monocytes with TLR ligands (LPS, PAM2 (PAM_2_CSK_4_), PAM3 (PAM_3_CSK_4_), and TB whole cell lysate), we were unable to detect IL-12p70 in cellular supernatants (Figure S2). In contrast, IL-10 was readily detectable, although at levels substantially lower than IL-6. To determine whether TLR1 and TLR6 polymorphisms were associated with IL-10 secretion, we stimulated PBMCs from 47 individuals with known TLR1_T1805G and TLR6_C745T genotypes. We stratified individuals into those who had hypofunctional genotypes for both TLR1 and TLR6 (TLR1/6_low represented by TLR1_1805GG and TLR6_745TT/CT) versus those with wild-type functional genotypes for both genes (TLR1/6_high represented by TLR1_1805GT/TT and TLR6_745CC). TLR1/6_low individuals had decreased levels of PAM2 and PAM3-induced IL-6 in comparison to TLR1/6_high individuals (Figure 6A). The TLR1/6_low individuals had lower PAM3-induced IL-10 levels in comparison to TLR1/6_high (Figure 6B). PAM2-stimulated IL-10 levels did not differ, although the level of induction was minimal. Interestingly, the TB-induced IL-6, but not IL-10, levels were lower in the TLR1/6_low group when compared to TLR1/6_high (Figure 6A and B). As a control, LPS-induced levels did not differ for IL-6 or IL-10. IL-12p70 was not detectable in any of the samples. We next examined the association of the individual polymorphisms with cytokine levels. Due to linkage disequilibrium between the 2 polymorphisms, the individual SNP analysis does not completely distinguish the separate effects of each polymorphism (R^2^ = 0.26; D' = 0.81), Similar to our previous studies, we found that genotypes TLR1_1805GG and TLR6_745CT/TT were associated with lower IL-6 levels in comparison to genotypes TLR1_T1805GT/TT and TLR6_745CC (Figure 6C and E) [27], [33]. We also found that genotype TLR1_1805GG was associated with lower IL-10 production in comparison to TLR1_1805GT/TT (Figure 6D). In contrast, TLR6_C745T genotypes were not associated with IL-10 levels (Figure 6F). As a control, these 2 polymorphisms were not associated with LPS-induced IL-6 secretion. Together, these data suggest that genetic regulation of monocyte production of IL-10 by TLR1/6 genetic variants (with the primary association probably due to TLR1_T1805G) provides a possible mechanism for the alteration of IFN-γ production by T-cells in BCG-vaccines.

## Discussion

The main finding of this study is that 4 well-defined hyporesponsive TLR polymorphisms are associated with increased *ex vivo* BCG-induced whole blood IFN-γ or IL-2 responses 10 weeks after *in vivo* BCG vaccination of newborns. To our knowledge, this is the first description of polymorphisms in innate pathway genes that affect the adaptive response to *in vivo* vaccination against a bacterial pathogen in humans. Various *in utero* and postpartum factors are likely to influence the immune response to BCG vaccination in newborns, including nutritional status, immunosuppression, antigen sensitization, and exposure to environmental mycobacteria. This study suggests that host genetic factors are associated with BCG vaccine responsiveness. Our findings are unlikely to be confounded by other factors as all infants were HIV-negative with similar nutritional status with exclusion of infants who had acute or chronic illnesses. Although evidence indicates that environmental bacteria modulate the immune response to BCG vaccination [4], [42], this variable is unlikely to confound our results since the infants received BCG at birth and would have minimal additional exposure during the 10 week follow-up. Previous studies indicate that BCG vaccination induces a potent Th1 response to mycobacterial antigens in newborns [43] with a wide range of IFN-γ levels [18]. Our data suggest that these previous observations may be partially attributable to variation in TLR1 or TLR6 and that host genetics are an important variable influencing the immune response to BCG vaccination.

One consistent and important pattern of association was that the hyporesponsive TLR1 or TLR6 allele was associated with increased IFN-γ or IL-2 levels after BCG vaccination. The molecular and cellular mechanisms by which these polymorphisms are having opposing effects on cytokine production are only partially understood. At the molecular level, each of the SNPs that had correlations with BCG responses in this study have previously been associated with *decreased* responsiveness to stimulation with purified TLR ligands in innate immune signaling assays (primary monocytes or transfected cell signaling assays). For example, we and others have shown that TLR1_T1805G regulates signaling in response to lipopeptide stimulation and essentially defines TLR1 deficiency in humans [26], [28], [34], [38], [44]. Studies of TLR1_1805GG individuals offer study design advantages similar to using *Tlr1-/-* mice, while avoiding the limitations of the mouse model. The second TLR1 polymorphism that was found to be associated with increased production of Th1 cytokines was A1188T, a non-synonymous SNP that results in an amino acid change from histidine to leucine (H305L). This SNP has not been as well characterized, but was shown in one study to be associated with partially reduced signaling after stimulation with synthetic lipopeptides in transfected cells [41]. We recently found that TLR6_C745T and TLR6_G1083C were associated with decreased IL-6 in response to lipopeptide stimulation in a whole blood cytokine assay. In addition, the TLR6_745T variant mediated less NF-κB signaling in a transfected HEK cells in response to stimulation with di-acylated lipopeptide or Mtb cell lysate. These findings correspond with our TLR1 results, where hyporesponsive variants in innate assays are associated with hyperresponsive Th1 T-cell cytokine production after BCG vaccination. The functional effect of the synonymous SNP G1083C is not currently known. This variant may be in linkage disequilibrium with a regulatory or coding region non-synonymous polymorphism, or it could directly regulate TLR6 mRNA expression.

The cellular mechanism underlying the association of TLR1 and TLR6-deficiency with increased Th1 T cell cytokine responses is also not known, but is likely secondary to effects on antigen presenting cells (APCs) such as monocytes, macrophages, and dendritic cells and their interactions with T cells. Alternative mechanisms include stimulation of TLRs on other cells such as neutrophils, T cells, or stromal cells at the vaccination site. Modulation of T-cell function by APCs could occur through several mechanisms including cytokine effects, genetic regulation of signaling pathways, and the activation of distinct immune responses by different innate immune receptors. Monocyte and dendritic cell-derived IL-12 induces and maintains the polarization of IFN-γ producing TH1 T-cells [45]. In contrast, IL-10 is an anti-inflammatory cytokine with pleiotropic effects including inhibition of IL-12 secretion and TH1 polarization [46], [47]. The ratio of IL-10 and IL-12 produced by APCs could influence the induction and/or maintenance of a TH1 polarized response. We found that PBMCs and monocytes secreted IL-10, but not IL-12p70 after stimulation with a panel of TLR ligands. In addition, TLR1/6 deficiency was associated with lower IL-10 levels. The genetic regulation of IL-10 levels in the absence of IL-12 provides a plausible mechanism whereby APC TLR1/6 deficiency could be associated with an increased TH1 T-cell response. Our *ex vivo* experimental conditions model a limited portion of the *in vivo* vaccination environment which includes multiple sites (e.g. vaccination site, lymph node, spleen, peripheral blood), various inflammatory milieus (with mixture of pro and anti-inflammatory cytokines from multiple cell types), and complex kinetics. Nevertheless, our *ex vivo* data demonstrates how TLR1/6 deficiency could promote TH1 T-cell polarization under some conditions.

In addition to genetic regulation of cytokine responses, the immune response is influenced by the specific innate immune receptors that are stimulated. Murine studies indicate that TLRs regulate cytokine production and maturation of DCs with subsequent modulation of T cell responses [48] [49] [50]. The degree and type of response (Th1 vs. Th2 vs. Th17) is modulated by which innate immune receptor is activated, including stimulation of Th1 responses by TLR4,7,8, and 9, Th2 responses by TLR2, and Th17 responses by NOD2. Our data is consistent with the murine studies which show that lack of TLR2 stimulation (via its heterodimeric partner TLR1 or TLR6) may promote an enhanced Th1 response. Mtb stimulates cells through several innate immune receptors, including TLR1,2,6,9, NOD2, DC-SIGN, CD43, Mincle, Marco and possibly TLR4, 8 and Dectin-1 [10], [51], [52], [53], [54], [55], [56]. With this diverse repertoire of receptors, several distinct T cell phenotypes may be promoted. Future identification of human deficiencies in other receptors will illuminate the mechanisms of innate immune modulation of T-cell responses and holds potential for rational selection of adjuvants.

There are several potential limitations of our study. Although the *ex vivo* BCG stimulation whole blood cytokine assay offers advantages of convenience and reproducibility for use in a large scale field trial, the cellular source of IFN-γ or IL-2 production is not identified with this method. In addition, the response is not antigen-specific due to the complex composition of BCG with both T cell antigens and ligands of innate immune receptors. We chose to examine IFN-γ, IL-2, and IL-13 since they are predominantly produced by T, NK, or NKT cells rather than monocytes, dendritic cells, neutrophils, or B cells. Previous studies by our group and others suggest that IFN-γ is almost exclusively made by T cells rather than NK or NKT cells in this assay [57], [58]. While this study focused on TLR-mediated immune responses to BCG vaccination, several addition PRRs that recognize mycobacterial antigens were not investigated including NOD2, DC-SIGN, Mincle (CLEC4E), dectin-1, and the mannose receptor [10]. This study also has numerous strengths including sample size, the presence of distinct discovery and validation sample sets, a standardized *in vivo* vaccination protocol given at a uniform age, and a consistent follow-up time point for assessing immune responses. To our knowledge, the combination of these study design features is novel and illuminates a unique role for TLR deficiencies in BCG vaccine responsiveness.

There are several potential impacts these findings may have on the development of novel diagnostics and prevention efforts for TB. For example, Mtb-specific T cell responses (PPD skin testing and more recently Interferon-Gamma Release Assays) have been used for over 100 years for diagnosing latent and active TB. Our observations are consistent with those of Cobat et al and support the concept that the genetic background of patients influences the results of immunodiagnostic tests and that alternative assays may need to be developed that are not dependent on host genetic variation [12], [13]. As an extreme example, the G allele of polymorphism TLR1_T1805G is rare in Asia and Africa, yet the most common allele in Europe and North America [27], [44]. Moreover, these results could potentially affect how we test vaccine efficacy in clinical trials. Because IFN-γ responses are often used to measure immunity to new vaccines in clinical trials, genetic differences may confound measurements of efficacy in such trials. Genotypes may need to be considered when analyzing efficacy data and our results could also potentially identify risks for vaccine failure.

Children with Mendelian disorders associated with an inability to produce or respond to IFN-γ (i.e. mutations in IL-12R, IL-12p40, and IFNγR1/R2) are highly susceptible to disseminated mycobacterial infection [59]. Our data describes a new genetic mechanism that could modulate IFN-γ levels in response to *Mycobacteria*. Although we do not currently know if these polymorphisms are associated with altered susceptibility to pediatric TB in our population, ongoing studies will address this question. Furthermore, recent work from our group and others suggests that TLR1-deficiency is clinically relevant with an association in several studies with susceptibility to leprosy and leprosy reversal reaction [26], [28], [44]. Further investigation is also warranted to address the kinetics of immune responses in BCG vaccinated infants as in this study we were limited by only one time-point and it is possible that variation is not sustained or that immunity may wane.

In summary, we examined BCG-induced cytokine responses in vaccinated infants and found that IFN-γ and IL-2 responses are dependent upon the genetic background of study participants. Taken together, these findings suggest that defects in innate pathway genes modulate adaptive responses to pathogens by altering the production of BCG-induced cytokines by T cells.

## Methods

### Ethics statement

All protocols for this study were approved by the Research Ethics Committee of the University of Cape Town and the Institutional Review Boards at the University of Washington and University of Medicine and Dentistry of New Jersey. Ethical guidelines of the US Department of Health and Human Services and the South African Medical Research Council were adhered to, including written informed consent from parents of study participants.

### Ligands and antigens

Ultrapure lipopolysacharide (LPS, TLR4 ligand, used in whole blood assays at 10 ng/mL, concentration in other assays mentioned below) isolated from *Salmonella minnesota* R595 was obtained from List Biological Labs, Inc. (Campbell, CA, USA). The lipopeptides PAM2 (PAM2CSKKKK, *S*-[2,3-bis(palmitoyloxy)-propyl]-(*R*)-cysteinyl-(lysyl)3-lysine, TLR2/6 ligand), and PAM3 (Pam3CSKKKK, *N*-palmitoyl-*S*-[2,3-bis- (palmitoyloxy)-propyl]-(*R*)-cysteinyl-(lysyl)3-lysine, TLR2/1 ligand) were synthetic lipopeptides obtained from EMC Microcollections (Tuebingen, Germany). Lysate from Mtb strain H37Rv (25 µg/mL) was obtained from J. Belisle (Colorado State University, Fort Collins, CO, USA; NIAID reagent contract). Lyophilized live Bacille Calmette-Guerin (BCG, 20 x 10^6^ CFU/mL) was obtained from Statens Serum Institute (Copenhagen, Denmark).

### Study population

Healthy infants, routinely vaccinated with intradermal BCG (Danish strain 1331; Statens Serum Institut, Copenhagen, Denmark) at birth, were enrolled from the South African Tuberculosis Vaccine Initiative (SATVI) field site in the Worcestor region, a rural area outside Cape Town, South Africa. HIV prevalence is very low in this region, while TB incidence is among the highest in the world [58]. Infants were excluded if known to be HIV-infected (upon rapid testing at enrollment site) or born to HIV-infected mothers. Infants with suspected or confirmed TB and those in contact with adults with pulmonary TB were also excluded as were infants with any other acute or chronic diseases or clinically apparent anemia at the time of enrollment. Suspected TB cases were referred to the SATVI clinical center where they were evaluated for diagnosis. Other exclusion criteria included infants who did not receive the BCG vaccination within 48 hours of birth, significant perinatal complications in the infant, and low birth weight infants (<2.5 kg). The overall ethnic distribution in this region is approximately 80% South African mixed ethnicity (genetically influenced by Malaysian, Indonesian, European Caucasoid and black African ethnic groups), 20% black African, and less than 1% Caucasian.

### Blood collection and stimulation and cryopreservation

At 10 weeks of age, heparinized blood was collected from BCG-vaccinated infants and 1mL was incubated *ex vivo* with 1.2×10^6^ CFU of BCG (Danish strain 1331). None of the infants in this study had active tuberculosis at the time of their 10 week blood draw or during 2 years of follow-up observation. Cytokine production was assessed in plasma harvested after a 7 hour incubation of whole blood with or without BCG. IFN-γ, IL-2 and IL-13 levels were measured by multiplex bead array technology according to manufacturer's instructions (Bio-Rad, Hercules, CA, USA) and read on a luminometer (Luminex, Austin, TX, USA). The range of detection for all cytokines was 2-32,000 pg/mL. Basal cytokine levels measured in plasma harvested from unstimulated blood were subtracted from values obtained from BCG-stimulated blood. Cells were also harvested at 72 hours and cryopreserved for T cell cytotoxicity and proliferation assays.

### Cytokine production in PBMCs after stimulation with purified TLR ligands

Cytokine assays were performed on PBMCs from infants in the South Africa vaccine cohort as well as adult healthy volunteers in Seattle. Study participants in Seattle were healthy adults with no known history of unusual susceptibility to infections. Peripheral blood mononuclear cells (PBMCs) were isolated from whole blood separated by centrifugation on a Ficoll-Hypaque gradient, plated at a density of 1×10^5^ cells per well in 96-well plates in RPMI (Invitrogen, Carlsbad, CA) +10% FBS (Invitrogen, Carlsbad, CA), and incubated overnight. For cells from South Africa (n = 87), cells were stimulated for 24 hours with 250 ng/mL of PAM2CysK4 and PAM3CysK3or ultrapure LPS at 100 ng/ml. For cells from Seattle adult donors (n = 47), PBMCs were stimulated for 24 hours immediately after isolation with media, 250 ng/mL PAM_2_CSK_4_, 250 ng/mL PAM_3_CSK_4_ 10 ng/ml LPS or 25 ucg/mL *Mycobacterium tuberculosis* (MTB) whole cell lysate. Supernatants were harvested and cytokines were measured using a sandwich ELISA (R&D Systems, Minneapolis, MN).

### T cell functional assays

Cryopreserved cells were thawed and incubated with 1 mL 1X Perm/Wash solution (BD Biosciences) for 10 minutes at room temperature to permeabilize cells. Cells were then washed and stained for 1 hr before samples were analyzed on a BD LSRII flow cytometer (BD Biosciences). For proliferation assays, cells were stained with CD4-PerCP (SK3), CD8-PerCPCy5.5 (SK1) and PE (B56) from BD Biosciences and CD3-QDot 605 (UCHT1) (Invitrogen). For cytotoxicity assays, cells were stained with CD3-Pacific Blue, CD4-Qdot 655, Ki67-PE, Perforin-APC, Granzyme B-Alexa Fluor 700 and Granulysin-Alexa Fluor 488, CD27-Qdot 655, CD28-PerCP-Cy5.5.

### Genomic techniques

PBMC isolated from study participants in South Africa were sent to Seattle, WA lab, where genomic DNA was obtained by purification using the QIAamp DNA blood kit (Qiagen) according to manufacturer's instructions. Genotyping of DNA was performed using a chip-based MALDI-TOF MassARRAY technique (Sequenom) as previously described. SpectroDESIGNER software was used to determine probes to be used for multiplex SNP assays; 5 ng of DNA was amplified in a 384-well plate following Sequenom's specifications. Shrimp alkaline phosphatase was added after PCR to prevent further incorporation of unused dNTPs that might interfere with the primer extension step. Next, allelic discrimination reactions were performed by adding a mixture of dNTPs and dideoxy NTPs to each well. MassEXTEND clean resin was added to the mixture to remove extraneous salts that could interfere with the MALDI-TOF analysis. Genotypes were determined by spotting 15 nL of each reaction onto a 386 SpectroCHIP (Sequenom), which was subsequently read by the MALDI-TOF mass spectrometer. For our primary analysis, we genotyped 9 well-characterized TLR-pathway SNPs which are associated with altered TLR gene function and/or susceptibility to different infections. The polymorphisms include TLR1_G1805T (amino acid (AA) change S602I, rs5743618), TLR2_T597C (AA N199N, rs3804099), TLR2_G2258A (AA R753Q, rs5743708), TLR4_A896G (AA D299G, rs4986790), TLR4_C1196T (AA T399I, rs4986791), TLR6_C745T (AA P249S, rs5743810), TLR6_G1083C (AA T361T, rs3821895) TIRAP_C539T (AAS180L, rs8177374), and TIRAP_C558T (AA A186A, rs7932766). Polymorphism TLR2_G2258A had only one heterozygote and was not further analyzed due to lack of variation.

### Statistical analysis

We examined whether polymorphisms were associated with cytokine levels with an allelic trend test using a general linear model (GLM) and results were expressed as mean +/− standard error of the mean. In the allelic trend test (also called a log-additive model) common homozygous genotypes (00) were assigned a value of 0, heterozygotes (01) a value of 1, and minor homozygous genotypes (11) a value of 2. The primary analysis examined whether 9 functionally characterized polymorphisms were associated with cytokine levels in a discovery sample set (n = 240). To avoid a Type I error from multiple comparisons, we analyzed initial findings in a validation sample set of the same size. Subjects were randomly assigned to discovery and validation sample sets using SPSS statistical software (SPSS for Windows, Rel. 15.0.1. 2006. Chicago: SPSS Inc.) As a secondary analysis, we also examined the combined dataset (n = 480) to identify associations involving low frequency polymorphisms. Two-sided testing was used for all comparisons to evaluate statistical significance, considering a P-value of ≤0.05 as significant. All analyses were performed using Stata (Intercooled Version 10.1, Statacorp LP; College Station, TX, USA). Cytokine data from TLR-stimulated PBMCs was analyzed with a Mann-Whitney U test due to the smaller sample size.

All SNPs analyzed were in Hardy Weinberg equilibrium with a P value cutoff of <0.001 in the South African Mixed Ancestry group indicating that there were no genotyping errors or major effects of population heterogeneity. To verify that our significant findings were not due to population admixture, we also performed South African Mixed Ancestry subgroup analyses. Stata program PWLD was used to calculate R^2^ and D' as measurements of linkage disequilibrium between the polymorphisms. To identify haplotype tagging SNPs, we examined data from the YRI (Yorubans in Nigeria) and CEU (Utah residents with European ancestry) populations from the International HapMap Project (http://www.hapmap.org) and other public databases with the Genome Variation Server (http://www.ncbi.nlm.nih.gov/SNP/ and www.innateimmunity.net). We searched a region on chromosome 4, 50 kilobases upstream and downstream of genes for TLR1, TLR6, TLR10, as well as TLR2 for tagged SNPs using an R^2^ cutoff of 0.8 for linkage disequilibrium and a minor allele frequency cut-off of 5%.

## Supporting Information

Figure S1 **TLR polymorphisms do not affect proliferation of T cells after** ***ex vivo*** **BCG stimulation.** Whole blood drawn 10 weeks after BCG at birth was restimulated with BCG *ex vivo* for 72 hours. Cell proliferation was measured in CD4-positive (A) and CD8-positive (B) T cells after *ex vivo* BCG re-stimulation of whole blood from infants vaccinated with BCG at birth. A general linear model was used to examine whether TLR polymorphisms were associated with proliferation after subtraction of unstimulated control values.
(TIF)

Figure S2 **TLR ligands induce IL-10, but not IL-12p70, in monocytes and PBMCs.** PBMCs and monocytes were isolated from 6 donors and stimulated for 20 hours with media, LPS (10 ng/mL), PAM_2_CSK_4_ (250 ng/mL), or PAM_3_CSK_4_ (250 ng/mL). Supernatant IL-10 (A) and IL-12p70 (B) levels were quantified by ELISA. IL-10, but not IL-12p70, was detectable in both PBMCs and monocytes.
(TIF)

Table S1 **Association of TLR-pathway polymorphisms with cytokine responses following bcg vaccination in discovery cohort.** Whole blood from 10-week old infants vaccinated at birth with BCG was re-stimulated with BCG ex vivo for 7 hours and plasma levels of IFN-γ, IL-2, and IL-13 were measured in a discovery cohort sample set (n = 240). P values were calculated from a general linear model that examined whether TLR polymorphisms were associated with BCG- induced cytokine levels after subtraction of unstimulated control values.
(DOCX)

Table S2 **Analysis of population admixture with 21 ancestry informative markers.** Population admixture was analysed using 21 ancestry informative markers. At each locus, χ2 represents Pearson's χ2 value for comparison of allele frequencies in groups of low (less than median) and high (greater than median) cytokine responses in the South African Mixed Ancestry Group. A global p value was calculated for the mean χ2 value for the 21 SNPs for each cytokine. For IFN-γ, IL-2, and IL-13, the global p values were 0.40, 0.38, and 0.39, respectively. Chrom, chromosomal location of SNP; Low, group of individuals with cytokine value below median; High, group of individuals with cytokine value above the median; HWE, Hardy-Weinberg Equilibrium; MAF, minor allele frequency.
(DOCX)

Table S3 **Association of polymorphisms in TLR1-6-10 locus with BCG-induced cytokine response.** Whole blood from 10-week old infants vaccinated at birth with BCG was re-stimulated with BCG ex vivo for 7 hours and plasma levels of IFN-γ, IL-2, and IL-13 were measured in a discovery cohort sample set (n = 240). P values were calculated from a general linear model that examined whether TLR polymorphisms were associated with BCG-induced cytokine levels after subtraction of unstimulated control values.
(DOCX)
