# Supplementary figures and images for: Association of Human TLR1 and TLR6 Deficiency with Altered Immune Responses to BCG Vaccination in South African Infants
Source: PLoS Pathog. 2011 Aug 11;7(8):e1002174. doi: 10.1371/journal.ppat.1002174 (PMC3154845; doi:10.1371/journal.ppat.1002174)

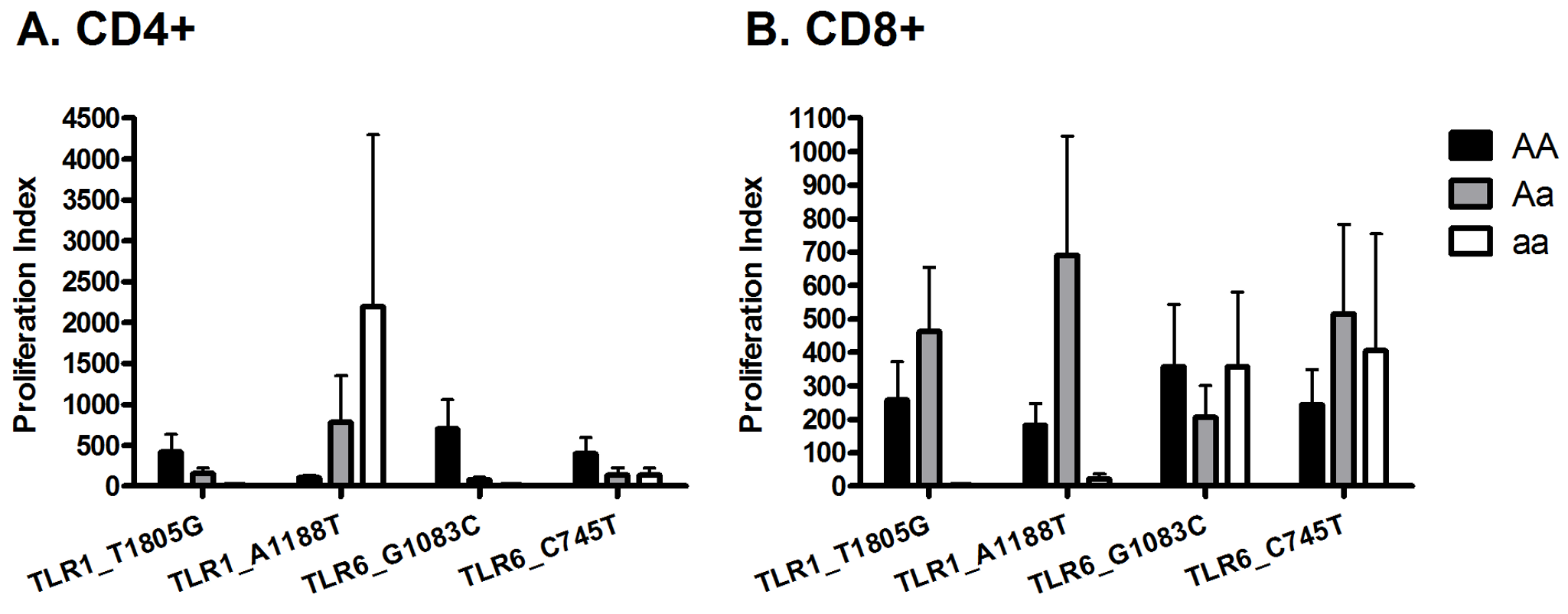

Supplement: Figure S1 — TLR polymorphisms do not affect proliferation of T cells after ex vivo BCG stimulation. Whole blood drawn 10 weeks after BCG at birth was restimulated with BCG ex vivo for 72 hours. Cell proliferation was measured in CD4-positive (A) and CD8-positive (B) T cells after ex vivo BCG re-stimulation of whole blood from infants vaccinated with BCG at birth. A general linear model was used to examine whether TLR polymorphisms were associated with proliferation after subtraction of unstimulated control values. (TIF) [file ppat.1002174.s001.tif]

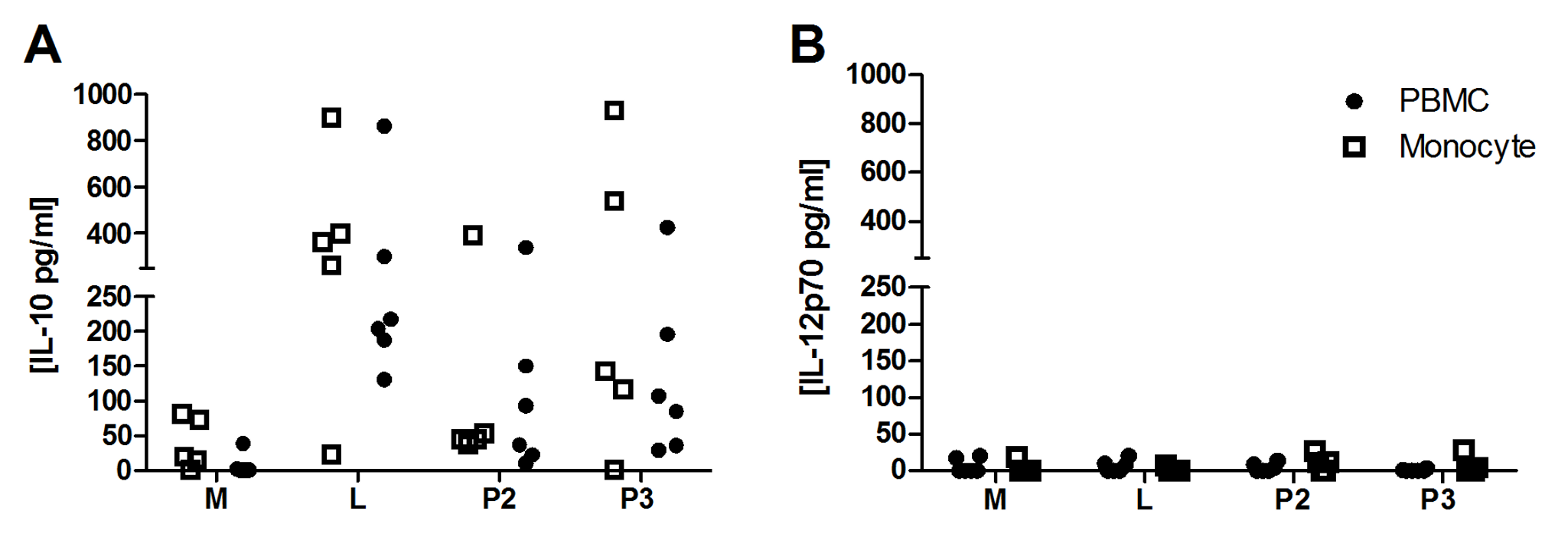

Supplement: Figure S2 — TLR ligands induce IL-10, but not IL-12p70, in monocytes and PBMCs. PBMCs and monocytes were isolated from 6 donors and stimulated for 20 hours with media, LPS (10 ng/mL), PAM2CSK4 (250 ng/mL), or PAM3CSK4 (250 ng/mL). Supernatant IL-10 (A) and IL-12p70 (B) levels were quantified by ELISA. IL-10, but not IL-12p70, was detectable in both PBMCs and monocytes. (TIF) [file ppat.1002174.s002.tif]
