# Supplementary material for: Association of Human TLR1 and TLR6 Deficiency with Altered Immune Responses to BCG Vaccination in South African Infants
Source: PLoS Pathog. 2011 Aug 11;7(8):e1002174. doi: 10.1371/journal.ppat.1002174 (PMC3154845; doi:10.1371/journal.ppat.1002174)
Supplement: Table S1 — Association of TLR-pathway polymorphisms with cytokine responses following bcg vaccination in discovery cohort. Whole blood from 10-week old infants vaccinated at birth with BCG was re-stimulated with BCG ex vivo for 7 hours and plasma levels of IFN-γ, IL-2, and IL-13 were measured in a discovery cohort sample set (n = 240). P values were calculated from a general linear model that examined whether TLR polymorphisms were associated with BCG- induced cytokine levels after subtraction of unstimulated control values. (DOCX) [file ppat.1002174.s003.docx]

|  |  |  |  | ***Combined Datasets*** | | | |  |  |
| --- | --- | --- | --- | --- | --- | --- | --- | --- | --- |
| **SNP** | **Genotype** | **Cytokine** |  | **N** | **Mean** | **SEM** | **P^b^** |  |  |
| **TLR6_C745T** | **C/C** | **IFN-γ** |  | 311 | 803.25 | 64.31 | **0.001** |  |  |
|  |  | **IL-2** |  | 310 | 535.67 | 37.45 | **0.030** |  |  |
|  |  | **IL-13** |  | 312 | 40.21 | 4.81 | 0.960 |  |  |
|  | **C/T** | **IFN-γ** |  | 54 | 1.4E+03 | 264.24 |  |  |  |
|  |  | **IL-2** |  | 53 | 698.86 | 103.93 |  |  |  |
|  |  | **IL-13** |  | 54 | 40.45 | 5.00 |  |  |  |
|  | **T/T** | **IFN-γ** |  | 6 | 1.9E+03 | 1.2E+03 |  |  |  |
|  |  | **IL-2** |  | 6 | 759.82 | 355.36 |  |  |  |
|  |  | **IL-13** |  | 6 | 36.33 | 12.78 |  |  |  |
| **TLR6_G1083C** | **G/G** | **IFN-γ** |  | 165 | 754.14 | 88.96 | **0.012** |  |  |
|  |  | **IL-2** |  | 165 | 464.12 | 38.90 | **0.009** |  |  |
|  |  | **IL-13** |  | 166 | 33.86 | 2.93 | 0.330 |  |  |
|  | **G/C** | **IFN-γ** |  | 173 | 926.87 | 105.95 |  |  |  |
|  |  | **IL-2** |  | 172 | 622.97 | 60.38 |  |  |  |
|  |  | **IL-13** |  | 173 | 46.65 | 8.24 |  |  |  |
|  | **C/C** | **IFN-γ** |  | 33 | 1.4E+03 | 283.15 |  |  |  |
|  |  | **IL-2** |  | 32 | 727.31 | 133.62 |  |  |  |
|  |  | **IL-13** |  | 33 | 37.37 | 6.64 |  |  |  |
| **TLR1_T1805G** | **T/T** | **IFN-γ** |  | 286 | 809.32 | 66.52 | **0.020** |  |  |
|  |  | **IL-2** |  | 285 | 542.50 | 39.80 | 0.466 |  |  |
|  |  | **IL-13** |  | 287 | 40.05 | 4.98 | 0.990 |  |  |
|  | **T/G** | **IFN-γ** |  | 82 | 1.3E+03 | 209.75 |  |  |  |
|  |  | **IL-2** |  | 81 | 6.4E+02 | 78.55 |  |  |  |
|  |  | **IL-13** |  | 82 | 41.93 | 6.55 |  |  |  |
|  | **G/G** | **IFN-γ** |  | 3 | 1275.08 | 246.67 |  |  |  |
|  |  | **IL-2** |  | 3 | 149.13 | 115.41 |  |  |  |
|  |  | **IL-13** |  | 3 | 16.38 | 12.83 |  |  |  |
| **TLR1_A1188T** | **A/A** | **IFN-γ** |  | 294 | 897.68 | 76.08 | **0.001** |  |  |
|  |  | **IL-2** |  | 293 | 574.73 | 41.57 | **0.009** |  |  |
|  |  | **IL-13** |  | 295 | 40.82 | 5.09 | 0.815 |  |  |
|  | **A/T** | **IFN-γ** |  | 73 | 723.43 | 103.90 |  |  |  |
|  |  | **IL-2** |  | 72 | 436.27 | 45.77 |  |  |  |
|  |  | **IL-13** |  | 73 | 35.58 | 3.59 |  |  |  |
|  | **T/T** | **IFN-γ** |  | 4 | 4.9E+03 | 2.0E+03 |  |  |  |
|  |  | **IL-2** |  | 4 | 1.7E+03 | 613.08 |  |  |  |
|  |  | **IL-13** |  | 4 | 60.84 | 20.68 |  |  |  |

**Table S1: South African Mixed Ancestry Subgroup Analysis of Association of TLR1 & 6 Polymorphisms with BCG-induced Cytokine Response^a^**

^a^Whole blood was drawn 10 weeks after BCG vaccination at birth and re-stimulated with BCG ex vivo for 7 hours and plasma levels of IFN-γ, IL-2, and IL-13 were measured.

^b^A general linear model was used to examine whether TLR polymorphisms were associated with BCG-induced cytokine levels after subtraction of unstimulated control values. Data shown represents subjects from the South African Mixed Ancestry Group.
