# Supplementary material for: Association of Human TLR1 and TLR6 Deficiency with Altered Immune Responses to BCG Vaccination in South African Infants
Source: PLoS Pathog. 2011 Aug 11;7(8):e1002174. doi: 10.1371/journal.ppat.1002174 (PMC3154845; doi:10.1371/journal.ppat.1002174)
Supplement: Table S2 — Analysis of population admixture with 21 ancestry informative markers. Population admixture was analysed using 21 ancestry informative markers. At each locus, χ2 represents Pearson's χ2 value for comparison of allele frequencies in groups of low (less than median) and high (greater than median) cytokine responses in the South African Mixed Ancestry Group. A global p value was calculated for the mean χ2 value for the 21 SNPs for each cytokine. For IFN-γ, IL-2, and IL-13, the global p values were 0.40, 0.38, and 0.39, respectively. Chrom, chromosomal location of SNP; Low, group of individuals with cytokine value below median; High, group of individuals with cytokine value above the median; HWE, Hardy-Weinberg Equilibrium; MAF, minor allele frequency. (DOCX) [file ppat.1002174.s004.docx]

**Table S2.** **Analysis of Population Admixture with 21 Ancestry Informative Markers^a^**

| **#** | **Chrom** | **rs #** | **HWE** | **IFN-γ** | | | | **IL-2** | | | | **IL-13** | | | |
| --- | --- | --- | --- | --- | --- | --- | --- | --- | --- | --- | --- | --- | --- | --- | --- |
|  |  |  |  | **Low** | **High** | **χ2** | **P** | **Low** | **High** | **χ2** | **P** | **Low** | **High** | **χ2** | **P** |
|  |  |  |  | **MAF** | **MAF** |  |  | **MAF** | **MAF** |  |  | **MAF** | **MAF** |  |  |
| 1 | 2 | 1028184 | 0.013 | 0.34 | 0.37 | 1.03 | 0.31 | 0.34 | 0.37 | 0.71 | 0.40 | 0.35 | 0.37 | 0.36 | 0.55 |
| 2 | 19 | 11672183 | 0.241 | 0.13 | 0.12 | 0.35 | 0.55 | 0.11 | 0.13 | 0.63 | 0.43 | 0.13 | 0.11 | 0.76 | 0.38 |
| 3 | 6 | 12665321 | 0.099 | 0.14 | 0.14 | 0.06 | 0.81 | 0.12 | 0.15 | 1.31 | 0.25 | 0.13 | 0.15 | 0.70 | 0.40 |
| 4 | 11 | 12785524 | 0.428 | 0.42 | 0.35 | 3.51 | 0.06 | 0.41 | 0.37 | 1.04 | 0.31 | 0.38 | 0.39 | 0.07 | 0.79 |
| 5 | 16 | 1364198 | 0.082 | 0.24 | 0.18 | 3.83 | 0.05 | 0.22 | 0.20 | 0.56 | 0.45 | 0.21 | 0.21 | 0.01 | 0.91 |
| 6 | 8 | 1380229 | 0.468 | 0.33 | 0.33 | 0.01 | 0.91 | 0.35 | 0.32 | 0.95 | 0.33 | 0.36 | 0.31 | 1.93 | 0.16 |
| 7 | 15 | 1433456 | 0.764 | 0.22 | 0.22 | 0.00 | 0.98 | 0.21 | 0.23 | 0.39 | 0.53 | 0.21 | 0.22 | 0.10 | 0.75 |
| 8 | 5 | 1479067 | 0.213 | 0.26 | 0.28 | 0.40 | 0.53 | 0.26 | 0.28 | 0.61 | 0.43 | 0.26 | 0.28 | 0.66 | 0.42 |
| 9 | 9 | 1566838 | 0.040 | 0.49 | 0.47 | 0.41 | 0.52 | 0.48 | 0.47 | 0.04 | 0.84 | 0.48 | 0.47 | 0.01 | 0.94 |
| 10 | 16 | 1582598 | 0.852 | 0.33 | 0.31 | 0.47 | 0.49 | 0.34 | 0.29 | 2.28 | 0.13 | 0.32 | 0.31 | 0.07 | 0.79 |
| 11 | 21 | 169479 | 0.945 | 0.14 | 0.13 | 0.10 | 0.75 | 0.16 | 0.11 | 3.14 | 0.08 | 0.12 | 0.15 | 1.25 | 0.26 |
| 12 | 20 | 2024628 | 0.006 | 0.48 | 0.45 | 0.66 | 0.42 | 0.47 | 0.47 | 0.01 | 0.94 | 0.48 | 0.46 | 0.47 | 0.50 |
| 13 | 4 | 2048022 | 0.185 | 0.39 | 0.41 | 0.33 | 0.56 | 0.38 | 0.41 | 0.82 | 0.37 | 0.37 | 0.42 | 2.06 | 0.15 |
| 14 | 7 | 327747 | 0.654 | 0.30 | 0.27 | 0.57 | 0.45 | 0.29 | 0.28 | 0.04 | 0.84 | 0.27 | 0.30 | 0.62 | 0.43 |
| 15 | 10 | 650389 | 0.566 | 0.14 | 0.16 | 1.01 | 0.31 | 0.14 | 0.16 | 0.68 | 0.41 | 0.15 | 0.15 | 0.01 | 0.94 |
| 16 | 12 | 695982 | 0.921 | 0.27 | 0.27 | 0.03 | 0.87 | 0.28 | 0.26 | 0.25 | 0.62 | 0.28 | 0.26 | 0.16 | 0.69 |
| 17 | 15 | 708682 | 0.389 | 0.13 | 0.10 | 1.09 | 0.30 | 0.12 | 0.11 | 0.21 | 0.65 | 0.12 | 0.11 | 0.23 | 0.63 |
| 18 | 17 | 807131 | 0.051 | 0.34 | 0.36 | 0.31 | 0.58 | 0.37 | 0.33 | 1.08 | 0.30 | 0.37 | 0.33 | 1.76 | 0.18 |
| 19 | 14 | 876287 | 0.733 | 0.44 | 0.44 | 0.00 | 0.99 | 0.45 | 0.43 | 0.29 | 0.59 | 0.40 | 0.48 | 4.18 | 0.04 |
| 20 | 13 | 914904 | 0.841 | 0.26 | 0.29 | 0.81 | 0.37 | 0.26 | 0.28 | 0.31 | 0.58 | 0.27 | 0.28 | 0.06 | 0.81 |
| 21 | 13 | 975423 | 0.480 | 0.39 | 0.37 | 0.19 | 0.66 | 0.36 | 0.40 | 0.98 | 0.32 | 0.38 | 0.38 | 0.02 | 0.89 |
|  |  |  |  |  |  |  |  |  |  |  |  |  |  |  |  |

**^a^**At each locus, χ2 represents Pearson’s χ2 value for comparison of allele frequencies in groups of low (less than median) and high

(greater than median) cytokine responses in the South African Mixed Ancestry Group. A global p value was calculated for the mean

χ2 value for the 21 SNPs for each cytokine. For IFN-γ, IL-2, and IL-13, the global p values were 0.40, 0.38, and 0.39, respectively.

Chrom, chromosomal location of SNP; Low, group of individuals with cytokine value below median; High, group of individuals with

cytokine value above the median; HWE, Hardy-Weinberg Equilibrium; MAF, minor allele frequency.
