# Supplementary material for: Association of Human TLR1 and TLR6 Deficiency with Altered Immune Responses to BCG Vaccination in South African Infants
Source: PLoS Pathog. 2011 Aug 11;7(8):e1002174. doi: 10.1371/journal.ppat.1002174 (PMC3154845; doi:10.1371/journal.ppat.1002174)
Supplement: Table S3 — Association of polymorphisms in TLR1-6-10 locus with BCG-induced cytokine response. Whole blood from 10-week old infants vaccinated at birth with BCG was re-stimulated with BCG ex vivo for 7 hours and plasma levels of IFN-γ, IL-2, and IL-13 were measured in a discovery cohort sample set (n = 240). P values were calculated from a general linear model that examined whether TLR polymorphisms were associated with BCG-induced cytokine levels after subtraction of unstimulated control values. (DOCX) [file ppat.1002174.s005.docx]

**Table S3: Association of polymorphisms in TLR1-6-10 locus with BCG-induced cytokine response^a^**

|  | | | ***Dataset 1*** | | |  | ***Dataset 2*** | | |  | ***Combined Datasets*** | | | |  |
| --- | --- | --- | --- | --- | --- | --- | --- | --- | --- | --- | --- | --- | --- | --- | --- |
| **SNP** | **Genotype** | **Cytokine** | **N** | **Mean** | **SEM** |  | **N** | **Mean** | **SEM** |  | **N** | **Mean** | **SEM** | **P ^a^** |  |
| **rs1039559** | T/T | **IFN-γ** | 154 | 819.94 | 97.61 |  | 148 | 771.19 | 83.84 |  | 302 | 796.05 | 64.45 | 0.111 |  |
|  |  | **IL-2** | 155 | 480.17 | 40.95 |  | 147 | 522.77 | 45.68 |  | 302 | 500.91 | 30.57 | 0.132 |  |
|  | T/C | **IFN-γ** | 68 | 1100.00 | 187.05 |  | 60 | 987.10 | 219.33 |  | 128 | 1000.00 | 142.45 |  |  |
|  |  | **IL-2** | 67 | 713.48 | 124.52 |  | 60 | 515.79 | 87.87 |  | 127 | 620.08 | 77.91 |  |  |
|  | C/C | **IFN-γ** | 2 | 1600.00 | 1500.00 |  | 10 | 837.74 | 258.48 |  | 12 | 959.97 | 295.73 |  |  |
|  |  | **IL-2** | 2 | 406.98 | 363.03 |  | 9 | 569.44 | 126.39 |  | 11 | 539.90 | 115.08 |  |  |
| **rs3775073** | G/G | **IFN-γ** | 114 | 816.42 | 120.08 |  | 103 | 755.23 | 102.55 |  | 217 | 787.37 | 79.53 | 0.080 |  |
|  |  | **IL-2** | 115 | 441.37 | 46.26 |  | 102 | 520.85 | 54.27 |  | 217 | 478.73 | 35.40 | 0.085 |  |
|  | G/A | **IFN-γ** | 99 | 929.60 | 124.64 |  | 91 | 959.27 | 160.22 |  | 190 | 943.81 | 100.27 |  |  |
|  |  | **IL-2** | 98 | 668.82 | 89.33 |  | 91 | 522.96 | 69.55 |  | 189 | 598.59 | 57.26 |  |  |
|  | A/A | **IFN-γ** | 13 | 1500.00 | 595.89 |  | 20 | 924.34 | 194.04 |  | 33 | 1200.00 | 261.97 |  |  |
|  |  | **IL-2** | 13 | 630.06 | 189.25 |  | 19 | 562.64 | 88.15 |  | 32 | 590.03 | 91.36 |  |  |
| **rs5743795** | G/G | **IFN-γ** | 201 | 873.95 | 88.81 |  | 188 | 850.62 | 94.32 |  | 389 | 862.68 | 64.60 | 0.592 |  |
|  |  | **IL-2** | 201 | 551.19 | 50.56 |  | 186 | 512.62 | 42.79 |  | 387 | 532.65 | 33.33 | 0.808 |  |
|  | G/A | **IFN-γ** | 25 | 1200.00 | 361.30 |  | 28 | 863.94 | 181.62 |  | 53 | 1000.00 | 194.83 |  |  |
|  |  | **IL-2** | 25 | 557.07 | 126.27 |  | 28 | 618.02 | 112.81 |  | 53 | 589.27 | 83.54 |  |  |
|  | A/A | **IFN-γ** | 1 | 518.42 | - |  | 2 | 558.92 | 558.78 |  | 3 | 545.42 | 322.89 |  |  |
|  |  | **IL-2** | 1 | 475.60 | - |  | 2 | 183.54 | 183.54 |  | 3 | 280.89 | 143.90 |  |  |
| **rs5743808** | T/T | **IFN-γ** | 185 | 969.60 | 104.67 |  | 179 | 853.20 | 84.15 |  | 364 | 912.36 | 67.37 | 0.192 |  |
|  |  | **IL-2** | 185 | 541.35 | 41.04 |  | 177 | 536.64 | 43.60 |  | 362 | 539.04 | 29.87 | 0.662 |  |
|  | C/T | **IFN-γ** | 39 | 642.46 | 115.97 |  | 34 | 924.82 | 313.77 |  | 73 | 773.97 | 158.38 |  |  |
|  |  | **IL-2** | 39 | 628.35 | 191.41 |  | 34 | 506.31 | 108.58 |  | 73 | 571.51 | 113.57 |  |  |
|  | C/C | **IFN-γ** | 2 | 642.15 | 509.97 |  | 5 | 214.62 | 103.79 |  | 7 | 336.77 | 154.05 |  |  |
|  |  | **IL-2** | 2 | 217.69 | 134.78 |  | 5 | 168.56 | 53.86 |  | 7 | 182.60 | 48.25 |  |  |
| **rs7665774** | C/C | **IFN-γ** | 136 | 819.45 | 108.09 |  | 131 | 746.77 | 88.66 |  | 267 | 783.79 | 70.07 | 0.100 |  |
|  |  | **IL-2** | 137 | 475.20 | 44.07 |  | 130 | 504.64 | 48.19 |  | 267 | 489.53 | 32.54 | 0.070 |  |
|  | G/C | **IFN-γ** | 85 | 1000.00 | 155.84 |  | 73 | 1000.00 | 188.67 |  | 158 | 1000.00 | 120.55 |  |  |
|  |  | **IL-2** | 84 | 671.15 | 99.59 |  | 73 | 549.07 | 77.75 |  | 157 | 614.38 | 64.38 |  |  |
|  | G/G | **IFN-γ** | 5 | 863.71 | 593.46 |  | 14 | 919.60 | 276.95 |  | 19 | 904.89 | 247.89 |  |  |
|  |  | **IL-2** | 5 | 690.28 | 492.27 |  | 13 | 564.17 | 123.19 |  | 18 | 599.20 | 154.15 |  |  |
| **rs7673348** | A/A | **IFN-γ** | 107 | 779.51 | 97.15 |  | 112 | 720.03 | 85.87 |  | 219 | 749.09 | 64.55 | 0.067 |  |
|  |  | **IL-2** | 107 | 459.63 | 43.89 |  | 111 | 513.27 | 53.47 |  | 218 | 486.94 | 34.69 | 0.164 |  |
|  | G/A | **IFN-γ** | 94 | 1100.00 | 173.67 |  | 75 | 963.76 | 181.99 |  | 169 | 1100.00 | 125.72 |  |  |
|  |  | **IL-2** | 94 | 672.20 | 95.24 |  | 75 | 520.24 | 73.39 |  | 169 | 604.76 | 62.29 |  |  |
|  | G/G | **IFN-γ** | 14 | 565.91 | 220.62 |  | 22 | 1100.00 | 318.06 |  | 36 | 919.03 | 215.38 |  |  |
|  |  | **IL-2** | 14 | 409.45 | 178.03 |  | 21 | 651.45 | 127.34 |  | 35 | 554.65 | 104.81 |  |  |
| **rs17616434** | C/C | **IFN-γ** | 135 | 839.59 | 108.50 |  | 139 | 759.65 | 85.34 |  | 274 | 799.04 | 68.71 | 0.114 |  |
|  |  | **IL-2** | 135 | 502.58 | 45.85 |  | 138 | 500.31 | 47.26 |  | 273 | 501.44 | 32.88 | 0.159 |  |
|  | T/C | **IFN-γ** | 80 | 1100.00 | 165.01 |  | 67 | 916.92 | 189.47 |  | 147 | 1000.00 | 124.32 |  |  |
|  |  | **IL-2** | 80 | 644.30 | 103.55 |  | 66 | 535.60 | 77.52 |  | 146 | 595.16 | 66.63 |  |  |
|  | T/T | **IFN-γ** | 12 | 546.80 | 250.22 |  | 12 | 1500.00 | 502.06 |  | 24 | 1000.00 | 292.36 |  |  |
|  |  | **IL-2** | 12 | 483.21 | 203.70 |  | 12 | 718.85 | 187.87 |  | 24 | 601.03 | 137.72 |  |  |
| **rs3923647** | A/A | **IFN-γ** | 172 | 898.32 | 97.87 |  | 181 | 845.80 | 92.41 |  | 353 | 871.39 | 67.14 | **<0.001** |  |
|  |  | **IL-2** | 172 | 568.01 | 57.04 |  | 180 | 539.14 | 45.50 |  | 352 | 553.25 | 36.26 | **0.002** |  |
|  | A/T | **IFN-γ** | 52 | 718.55 | 99.11 |  | 35 | 709.95 | 174.98 |  | 87 | 715.09 | 91.39 |  |  |
|  |  | **IL-2** | 52 | 419.98 | 48.14 |  | 34 | 418.09 | 71.85 |  | 86 | 419.23 | 40.41 |  |  |
|  | T/T | **IFN-γ** | 3 | 4600.00 | 2800.00 |  | 2 | 3300.00 | 2400.00 |  | 5 | 4100.00 | 1700.00 |  |  |
|  |  | **IL-2** | 3 | 1900.00 | 838.53 |  | 2 | 758.06 | 346.06 |  | 5 | 1400.00 | 546.92 |  |  |
| **rs3924112** | C/C | **IFN-γ** | 205 | 874.92 | 88.00 |  | 186 | 843.66 | 94.93 |  | 391 | 860.05 | 64.48 | 0.501 |  |
|  |  | **IL-2** | 205 | 546.51 | 49.70 |  | 185 | 510.06 | 42.78 |  | 390 | 529.22 | 33.05 | 0.494 |  |
|  | C/T | **IFN-γ** | 21 | 1200.00 | 411.83 |  | 33 | 850.43 | 164.95 |  | 54 | 999.39 | 188.64 |  |  |
|  |  | **IL-2** | 21 | 603.90 | 146.09 |  | 32 | 610.98 | 104.22 |  | 53 | 608.17 | 84.63 |  |  |
|  | T/T | **IFN-γ** | 1 | 518.42 | . |  | 1 | 1100.00 | . |  | 2 | 818.06 | 299.64 |  |  |
|  |  | **IL-2** | 1 | 475.60 | . |  | 1 | 367.08 | . |  | 2 | 421.34 | 54.26 |  |  |
| **rs4833095** | C/C | **IFN-γ** | 131 | 849.24 | 112.27 |  | 137 | 762.50 | 86.17 |  | 268 | 804.90 | 70.29 | 0.074 |  |
|  |  | **IL-2** | 132 | 492.83 | 45.62 |  | 136 | 494.76 | 47.73 |  | 268 | 493.81 | 32.98 | 0.060 |  |
|  | C/T | **IFN-γ** | 86 | 976.91 | 151.98 |  | 70 | 918.64 | 182.55 |  | 156 | 950.77 | 116.81 |  |  |
|  |  | **IL-2** | 85 | 627.53 | 99.01 |  | 69 | 551.65 | 74.85 |  | 154 | 593.53 | 64.00 |  |  |
|  | T/T | **IFN-γ** | 10 | 1100.00 | 382.05 |  | 11 | 1500.00 | 549.52 |  | 21 | 1300.00 | 335.91 |  |  |
|  |  | **IL-2** | 10 | 679.77 | 241.83 |  | 11 | 697.00 | 204.41 |  | 21 | 688.80 | 153.23 |  |  |
| **rs4321646** | C/C | **IFN-γ** | 77 | 980.29 | 147.94 |  | 56 | 1100.00 | 218.24 |  | 133 | 1000.00 | 125.15 | 0.392 |  |
|  |  | **IL-2** | 76 | 606.75 | 110.66 |  | 54 | 521.30 | 73.29 |  | 130 | 571.26 | 71.36 | 0.780 |  |
|  | C/T | **IFN-γ** | 96 | 854.64 | 126.90 |  | 112 | 662.32 | 87.72 |  | 208 | 751.08 | 75.35 |  |  |
|  |  | **IL-2** | 97 | 514.78 | 49.64 |  | 112 | 494.27 | 53.70 |  | 209 | 503.79 | 36.78 |  |  |
|  | T/T | **IFN-γ** | 54 | 893.55 | 206.52 |  | 53 | 891.49 | 156.72 |  | 107 | 892.53 | 129.35 |  |  |
|  |  | **IL-2** | 54 | 539.72 | 81.98 |  | 53 | 566.73 | 89.05 |  | 107 | 553.10 | 60.21 |  |  |
| **rs10856837** | G/G | **IFN-γ** | 187 | 932.20 | 102.53 |  | 173 | 897.84 | 99.27 |  | 360 | 915.69 | 71.41 | 0.217 |  |
|  |  | **IL-2** | 187 | 518.49 | 41.88 |  | 172 | 552.39 | 45.33 |  | 359 | 534.73 | 30.75 | 0.835 |  |
|  | G/A | **IFN-γ** | 34 | 858.12 | 162.62 |  | 44 | 649.12 | 149.91 |  | 78 | 740.22 | 110.27 |  |  |
|  |  | **IL-2** | 34 | 768.07 | 208.75 |  | 43 | 416.44 | 81.68 |  | 77 | 571.71 | 103.99 |  |  |
|  | A/A | **IFN-γ** | 2 | 314.99 | 15.56 |  | 2 | 690.06 | 690.06 |  | 4 | 502.52 | 301.87 |  |  |
|  |  | **IL-2** | 2 | 471.09 | 255.44 |  | 2 | 290.34 | 290.34 |  | 4 | 380.71 | 166.27 |  |  |
| **rs7694115** | G/G | **IFN-γ** | 76 | 907.10 | 157.85 |  | 73 | 845.09 | 143.69 |  | 149 | 876.72 | 106.62 | 0.785 |  |
|  |  | **IL-2** | 76 | 650.49 | 107.15 |  | 72 | 532.17 | 68.31 |  | 148 | 592.93 | 64.25 | 0.263 |  |
|  | A/G | **IFN-γ** | 107 | 929.47 | 136.41 |  | 112 | 863.22 | 125.53 |  | 219 | 895.59 | 92.35 |  |  |
|  |  | **IL-2** | 108 | 465.33 | 49.92 |  | 111 | 555.22 | 59.92 |  | 219 | 510.89 | 39.12 |  |  |
|  | A/A | **IFN-γ** | 41 | 833.22 | 151.91 |  | 35 | 785.37 | 170.58 |  | 76 | 811.18 | 112.79 |  |  |
|  |  | **IL-2** | 40 | 597.73 | 98.67 |  | 35 | 402.47 | 65.94 |  | 75 | 506.61 | 61.62 |  |  |

^a^Whole blood was drawn 10 weeks after BCG vaccination at birth and re-stimulated with BCG ex vivo for 7 hours and plasma levels of IFN-γ, IL-2, and IL-13 were measured.^b^A general linear model was used to examine whether TLR polymorphisms were associated with BCG-induced cytokine levels after subtraction of unstimulated control values. Data shown represents subjects from the South African Mixed Ancestry Group.
